# Gene swapping in the dead zone

**DOI:** 10.7554/eLife.04600

**Published:** 2014-10-13

**Authors:** Jillian Petersen, Nicole Dubilier

**Affiliations:** 1 **Jillian Petersen** is in the Symbiosis Department, Max Planck Institute of Marine Microbiology, Bremen, Germanyjmpeters@mpi-bremen.de; 2 **Nicole Dubilier** is in the Symbiosis Department, Max Planck Institute of Marine Microbiology, Bremen, Germanyndubilie@mpi-bremen.de

**Keywords:** SUP05, bacteriophages, viruses, single cell genomics, oxygen minimum zone, viral dark matter, viruses

## Abstract

Viruses can swap DNA between bacteria that live in regions of the oceans with little or no oxygen.

**Related research article** Roux S, Hawley AK, Torres Beltran M, Scofield M, Schwientek P, Stepanauskas R, Woyke T, Hallam SJ, and Sullivan MB. 2014. Ecology and evolution of viruses infecting uncultivated SUP05 bacteria as revealed by single-cell- and meta-genomics. *eLife* **3**:e03125. doi: 10.7554/eLife.03125

**Image** Schematic of two different viruses introducing new DNA into the genome of a marine bacterium

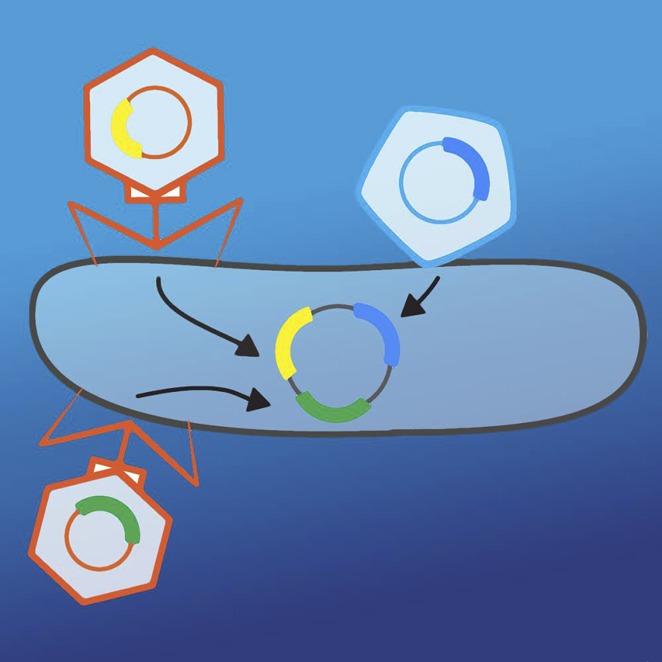

The oceans are home to a staggering number of viruses, roughly 10^30^, making them the most abundant organisms on the planet. Most of these viruses infect bacteria, which are also found in huge numbers. Genetic surveys have revealed that the viruses and bacteria in the oceans are extremely diverse, yet the interactions between them can be highly specific. However, it is challenging to link marine bacteria with their viruses because most of them cannot be cultured on artificial media in the lab, which is the traditional method for studying virus–bacteria interactions.

Now, in *eLife*, a collaboration between researchers from the US and Canada—with Simon Roux of the University of Arizona as first author—has used a new technology called single-cell genome sequencing to link a group of highly abundant and widespread marine bacteria with two specific groups of viruses, without having to culture either the host bacteria or the virus in the lab (Roux et al., 2014). The collaboration was led by Matthew Sullivan of Arizona and Steven Hallam of the University of British Columbia, and also included researchers at the Bigelow Laboratory for Ocean Sciences and the DOE Joint Genome Institute.

The bacteria, called SUP05, are marine bacteria that oxidize reduced sulfur compounds such as hydrogen sulfide to gain energy (Walsh et al., 2009). They are close relatives of the sulfur-oxidizing bacteria that live inside mussels and clams at marine hydrothermal vents and cold seeps (Lavik et al., 2008; Petersen et al., 2012). The energy they obtain from oxidizing sulfur is used to fix carbon dioxide to make the organic carbon compounds the bacteria need to grow.

SUP05 bacteria are widespread in open water and are particularly abundant in regions of water that contain very little oxygen. These oxygen minimum zones (OMZs), also known as marine dead zones, form where oxygen respiration is rapid, and the physical processes that would replenish oxygen supplies (such as mixing and exposure to the atmosphere) are weak. It is estimated that between 1 and 7% of the oceans are OMZs. These large swathes of water are lethal to animals that require oxygen, but they are hot-spots for microbes that can live on sulfur (Wright et al., 2012). SUP05 bacteria can survive in oxygen-poor environments because they use dissolved nitrate instead of oxygen for respiration.

The microbes living in OMZs transform sulfur, nitrogen and carbon on such large scales that they can alter ocean chemistry, and contribute to climate change through the production of greenhouse gases. In turn, global warming is predicted to increase the extent and intensity of OMZs (Stramma et al., 2008). Therefore, understanding the factors that control the activities of microbes in OMZs is crucial for predicting their role in future climate change.

Roux et al. sampled the water of Saanich Inlet, a fjord on the Canadian Pacific coast where an OMZ forms every summer. Samples were taken at three different depths that spanned the transition from oxygenated surface waters to the deeper low-oxygen ‘core’ of the OMZ. From these samples, individual bacterial cells were sorted into tiny wells by a device called a flow cytometer. The DNA in each well (including DNA from the viruses infecting the bacterial cells) was then copied to produce enough DNA to individually sequence the genome of each cell.

Roux et al. found that 33% of all the single-cell genomes contained viral DNA, the very first estimate of infection rates in a natural bacterial population. This closely matches previous estimates of whole-community infection rates based on metagenome sequencing, where the DNA of all the microbes in a sample is sequenced altogether (Suttle, 2007). Intriguingly, Roux et al. found that the percentage of infected SUP05 cells varied between samples taken at different depths, with the highest infection rates in the deepest part of the OMZ, where SUP05 cells are most abundant (Figure 1).

**Figure 1.**
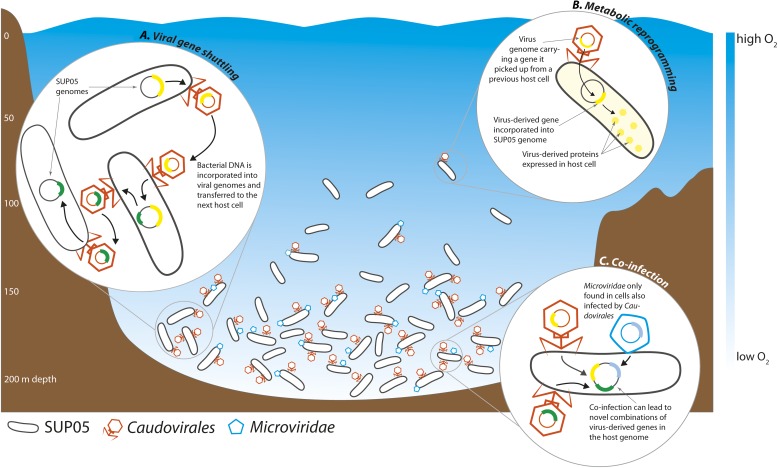
Interactions between viruses and bacteria in the Saanich Inlet marine ‘dead zone’. In summer, viruses from the order *Caudovirales* (orange) and the family *Microviridae* (blue) become abundant at depths where the water contains very little oxygen (pale blue regions) because their hosts, SUP05 bacteria (black), are also abundant in these oxygen minimum zones. The viruses can shuttle bacterial genes between different host cells (**A**). This can lead to changes in the metabolism of the host bacteria if the new genes are expressed by the host cells (**B**). Co-infection of a host cell by two different viruses could lead to new combinations of genes in the bacterial genome (**C**).

Understanding the specificity of viruses—that is, understanding which organisms they can infect—is essential for predicting their effects in nature, as shown by the recent outbreaks of Ebola and other viruses in humans. Unfortunately, very little is known about the specificity of most marine viruses. Roux et al. identified five new types of viruses from the order *Caudovirales* and the family *Microviridae* that infect SUP05 bacteria. Although it is not known whether these viruses can infect other bacterial species, the identification of one host species is a major step forward. The *Microviridae* were almost always found in cells infected by *Caudovirales*, which indicates that they may cooperate in some way.

Roux et al. then used the viral genomes to search through genomic databases for more information about their distribution in the environment, starting with the data they had collected from the waters of Saanich Inlet. Similar SUP05-infecting viruses were consistently found in the OMZ over the three-year sampling period. Roux et al. were also able to observe evolution in the viruses, with new genes that appeared in the genomes of some of the viruses in the second sampling year being found in virtually all of the viruses by the third year.

Next, they searched for the SUP05-infecting viruses in metagenomes—collections of all the genetic material found in environmental samples—from around the globe. The viruses that infect SUP05 in Saanich Inlet were rarely found in other ocean regions, even those adjacent to the Inlet, or in distant regions populated by similar SUP05 bacteria. This shows a surprisingly strict geographic restriction, despite regular seasonal mixing of Saanich Inlet with waters from the neighboring north eastern Pacific Ocean.

Viruses can alter the course of bacterial evolution by introducing new genes into the cells they infect. Bacterial DNA can be taken up by virus particles and shuttled to the next host bacterial cell, where it can be used to the new cell's advantage (Figure 1). Well-known examples of virus-mediated gene shuttling are the transfer of key photosynthesis genes between marine cyanobacteria, and the exchange of cholera toxin genes between different *Vibrio cholerae* strains (Faruque et al., 1998; Lindell et al., 2004). Genes for sulfur cycling were recently discovered in viruses associated with SUP05 from hydrothermal vents (Anantharaman et al., 2014). Roux et al. also discovered genes for sulfur oxidation in the genomes of SUP05 viruses from Saanich Inlet. Thus, viruses might play a key role in altering core metabolism of SUP05, not only in the deep sea, but also in widespread OMZs. The sharing of bacterial genes by viruses may be a universal feature of natural microbial communities.

Bacteria are often referred to as the engines that drive nutrient cycles in the marine environment. They do this by catalyzing key processes such as CO_2_ fixation, organic matter recycling, conversion of nitrogen, and removal of toxic hydrogen sulfide (Falkowski et al., 2008). To extend the analogy, viruses may be the mechanics who tinker with the efficiency of these engines by shuttling metabolic genes between bacterial host cells.
